# Sirtuin 6—A Key Regulator of Hepatic Lipid Metabolism and Liver Health

**DOI:** 10.3390/cells12040663

**Published:** 2023-02-19

**Authors:** X. Charlie Dong

**Affiliations:** 1 Department of Biochemistry and Molecular Biology, Indiana University School of Medicine, Indianapolis, IN 46202, USA; xcdong@iu.edu; 2 Center for Computational Biology and Bioinformatics, Indiana University School of Medicine, Indianapolis, IN 46202, USA

**Keywords:** SIRT6, lipogenesis, fatty acid oxidation, inflammation, fibrosis, hepatocellular carcinoma, modulator

## Abstract

Sirtuin 6 (SIRT6) is an NAD-dependent deacetylase/deacylase/mono-ADP ribosyltransferase, a member of the sirtuin protein family. SIRT6 has been implicated in hepatic lipid homeostasis and liver health. Hepatic lipogenesis is driven by several master regulators including liver X receptor (LXR), carbohydrate response element binding protein (ChREBP), and sterol regulatory element binding protein 1 (SREBP1). Interestingly, these three transcription factors can be negatively regulated by SIRT6 through direct deacetylation. Fatty acid oxidation is regulated by peroxisome proliferator activated receptor alpha (PPARα) in the liver. SIRT6 can promote fatty acid oxidation by the activation of PPARα or the suppression of miR-122. SIRT6 can also directly modulate acyl-CoA synthetase long chain family member 5 (ACSL5) activity for fatty acid oxidation. SIRT6 also plays a critical role in the regulation of total cholesterol and low-density lipoprotein (LDL)-cholesterol through the regulation of SREBP2 and proprotein convertase subtilisin/kexin type 9 (PCSK9), respectively. Hepatic deficiency of Sirt6 in mice has been shown to cause hepatic steatosis, inflammation, and fibrosis, hallmarks of alcoholic and nonalcoholic steatohepatitis. SIRT6 can dampen hepatic inflammation through the modulation of macrophage polarization from M1 to M2 type. Hepatic stellate cells are a key cell type in hepatic fibrogenesis. SIRT6 plays a strong anti-fibrosis role by the suppression of multiple fibrogenic pathways including the transforming growth factor beta (TGFβ)-SMAD family proteins and Hippo pathways. The role of SIRT6 in liver cancer is quite complicated, as both tumor-suppressive and tumor-promoting activities have been documented in the literature. Overall, SIRT6 has multiple salutary effects on metabolic homeostasis and liver health, and it may serve as a therapeutic target for hepatic metabolic diseases. To date, numerous activators and inhibitors of SIRT6 have been developed for translational research.

## 1. Introduction

**Figure 1:**
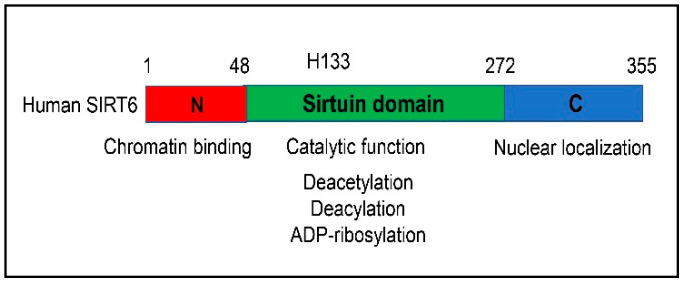
**Schematic diagram of the human SIRT6 protein domain structure.** SIRT6 protein can be divided into three domains: N-terminal, central, and C-terminal.

Sirtuin 6 (SIRT6) is one of the members of the class III histone deacetylase (HDAC) family, as they use a unique nicotinamide adenine dinucleotide (NAD) as a cofactor [1]. SIRT6 is one of three nuclear sirtuins, along with SIRT1 and SIRT7. The human SIRT6 gene spans a region of 8427 bp on chromosome 19, with nine exons and eight introns, encoded by the minus strand [2]. The SIRT6 gene transcription in the liver is regulated by fasting. SIRT1, nuclear respiratory factor 1 (NRF1), and forkhead box O3 (FOXO3) play a significant role in the activation of the SIRT6 gene transcription in the liver [3]. There are at least nine documented transcripts generated by alternative splicing according to the NCBI Gene annotations. The longest translation product has 355 amino acids. Biochemical analysis has delineated three main regions—N-terminal, central, and C-terminal (Figure 1) [4]. The central region has the well-conserved catalytic domain among the sirtuin family members. Three catalytic activities have been reported for the SIRT6 protein: deacetylation, deacylation, and mono-ADP-ribosylation [5,6,7,8,9,10]. A list of known substrates is described in Table 1. Acetylated histone H3 lysines 9 and 56 (K9 and K56) are two major histone substrates that can be deacetylated by SIRT6 [6,7,8]. Numerous non-histone deacetylation substrates including general control nonrepressed protein 5 (GCN5), X-box binding protein 1 (XBP1), Yes1-associated transcriptional regulator (YAP), WW domain-containing transcription regulator 1 (WWTR1/TAZ), SMAD family member 2 (SMAD2), and SMAD3 have been reported as well [11,12,13,14,15].

**Figure 2:**
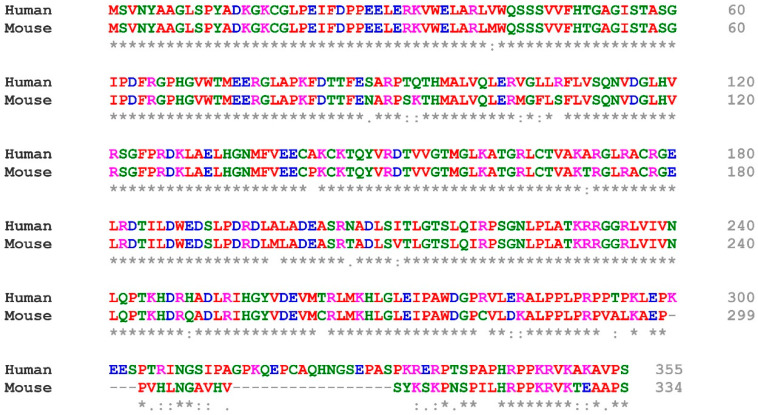
**Human and mouse SIRT6 peptide sequence alignments.** The longest isoforms of human and mouse SIRT6 proteins were aligned using the Clustal Omega multiple sequence alignment tool on the EMBL-EBI website.

In contrast, very few deacylation and ADP-ribosylation substrates have been reported so far. Tumor necrosis factor α (TNFα) and RAS-related 2 (R-Ras2) are two known deacylation substrates [9,10]. Poly (ADP-ribose) polymerase 1 (PARP1), tripartite motif-containing 28 (TRIM28/KAP1), lysine demethylase 2A (KDM2A), and SWI/SNF-related matrix-associated actin-dependent regulator of chromatin subfamily C member 2 (SMARCC2/BAF170) are among the known ADP-ribosylation substrates [63,64,65,67]. Four basic residues (K346, R347, K349, K351) in the C-terminal region are key to the SIRT6 nuclear localization. In addition to chromatin binding, the N-terminal region also facilitates the SIRT6 catalytic activities. Moreover, the catalytic region and the C-terminal region also contribute to the SIRT6 association with chromatin [4]. Human and mouse SIRT6 protein sequences share a high similarity in both the N-terminal and central regions but are very divergent in the C-terminal region, whereas the mouse Sirt6 protein has two missing fragments (4 and 17 amino acids, respectively) (Figure 2). It is unclear whether those sequence differences could affect their biological functions.

Gene knockout studies have suggested that SIRT6 is an essential gene for embryonic and organismal development. Sirt6 whole-body knockout (deletion of exon 1) mice on the 129S genetic background exhibit growth retardation and metabolic (hypoglycemia) and developmental defects (loss of subcutaneous fat, lordokyphosis, and lymphopenia) and die at about 4 weeks of age [68]. Another line of Sirt6 knockout mice (deletion of exons 2 and 3) on a mixed 129S/Black Swiss/FVB genetic background has an approximately 40% survival rate up to 1 year. SIRT6-deficient monkeys also exhibit severe developmental retardation in most tissues and organs analyzed and die hours after birth [69]. Although it has been reported that SIRT6 is involved in the maintenance of genome stability in rodents and cell lines, SIRT6 gene knockout in monkeys does not lead to either genome or epigenome instability. This discrepancy between rodents and monkeys requires further investigation. Several genetic variants in the human SIRT6 gene have been reported. The linked N308K and A313S dual variants have been identified in the Ashkenazi Jewish centenarians. This SIRT6 variant exhibits weaker deacetylase but stronger mono-ADP ribosyl transferase activities compared to the common SIRT6 variant, including enhanced lamin A/C (LMNA) ribosylation. Additionally, the N308K/A313S variant of SIRT6 has been shown to exert a stronger suppression of LINE1 retrotransposons, better function in DNA double-strand break repair, and higher killing of cancer cells (HT1080 fibrosarcoma cell line and Hela cervical cancer cell line) but not normal cells than the common SIRT6 variant [66]. The homozygous mutation of D63H in humans has been reported to be lethal perinatally. Several prenatal abnormalities have been observed, including intrauterine growth retardation, microcephaly, craniofacial anomalies, congenital heart defects, and sex reversal in male fetuses. The SIRT6 D63H mutant has very low deacetylase and demyristoylase activities. Mouse embryonic stem cells (mESCs) or human induced pluripotent stem cells (iPSCs) carrying homozygous D63H fail to differentiate into embryoid bodies due to unrepressed pluripotent gene expression [70]. The exome sequencing of 12 types of tumors from patients has revealed eight mutations in SIRT6. The mutations are found in non-small-cell lung cancer, renal clear cell carcinoma, cervical carcinoma, and melanoma. Seven mutations are missense and one mutation is nonsense, including D25N, E36V, D63Y, A89S, D116N, T263P, P274L, and E260Stop. D116N and E260Stop mutants have reduced levels in chromatin. D116N also had reduced protein stability. D25N, E36V, A89S, T263P, and P274L display approximately 50% deacetylase activity of WT SIRT6, whereas D116N and D63Y only exhibit about 2% of wild-type (WT) SIRT6 deacetylase activity. D25N, A89S, D116N, and E260Stop mutants display less of a tumor suppression effect than the WT SIRT6 in a xenograft mouse model [71].

## 2. SIRT6 in Hepatic Lipogenesis

**Figure 3:**
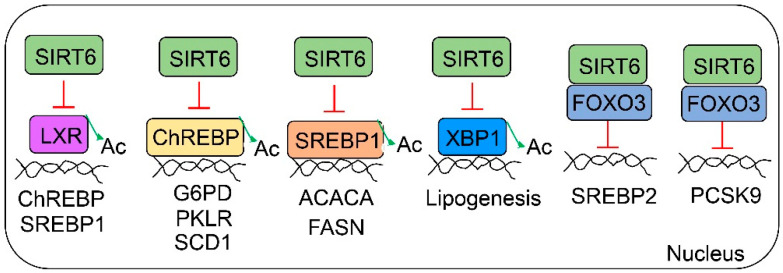
**Regulation of hepatic triglycerides and cholesterol by SIRT6.** Several master regulators for triglyceride biosynthesis, including LXR, ChREBP, SREBP1, and XBP1, can be suppressed by SIRT6 through deacetylation. SIRT6 can be recruited to the promoter of the SREBP2 gene through FOXO3 transcription factor to suppress hepatic cholesterol biosynthesis. Additionally, SIRT6 and FOXO3 also suppress the PCSK9 gene transcription to reduce LDL-cholesterol in the blood circulation.

As an epigenetic regulator, SIRT6 plays a critical role in the regulation of hepatic triglycerides, cholesterol, and low-density lipoprotein (LDL) homeostasis (Figure 3). For the de novo lipogenesis, SIRT6 can suppress at least four major transcription factors—liver X receptor (LXR), carbohydrate response element binding protein (ChREBP), sterol regulatory element binding protein 1 (SREBP1), and X-box binding protein 1 (XBP1) [15,20]. LXR is a nuclear receptor that can be activated by oxysterols (cholesterol derivatives) and functions with retinoid X receptor (RXR). Both SREBP1 and ChREBP can be transcriptionally activated by LXR. Interestingly, SIRT6 can repress LXR activity by the deacetylation of K432 and also directly represses SREBP1 and ChREBP by deacetylation at K289 and K672, respectively [20]. Liver-specific Sirt6 knockout (LKO) mice are more susceptible to Western diet-induced fatty liver disease, manifesting elevated serum and hepatic diacylglycerol and triglyceride levels compared to WT mice. Hepatic SREBP1 and ChREBP protein levels are significantly increased in Sirt6 LKO mice compared to those in WT mice, whereas LXRα/β protein levels are not significantly changed [20]. SIRT6 also regulates the SREBP1 transcriptional activity in a circadian manner through an interaction of core clock components—clock circadian regulator (CLOCK) and basic helix–loop–helix ARNT-like 1 (BMAL1) [72]. When Sirt6 is deleted in the mouse liver, the knockout mice develop hepatic steatosis on both normal chow and high-fat diets [3,20,73]. On a chow diet, nearly half of the Sirt6 LKO mice develop fatty liver at 5–6 months of age, and 90% of LKO mice have fatty liver by 13 months of age [3]. Multiple glycolytic and lipogenic genes, including glucokinase (GCK), pyruvate kinase (PKLR), acetyl-CoA carboxylase alpha (ACACA), fatty acid synthase (FASN), ELOVL fatty acid elongase 6 (ELOVL6), and stearoyl-CoA desaturase (SCD), are upregulated in the liver of Sirt6 LKO mice [3]. XBP1 also plays a critical role in hepatic lipogenesis, especially under ER stress conditions. Interestingly, SIRT6 can deacetylate XBP1 at the K257 and K297 residues. As a result, XBP1 undergoes ubiquitin-proteasome-mediated protein degradation [15]. As a matter of fact, SIRT6 protein is also subjected to ubiquitin-mediated degradation. The removal of ubiquitin modification from SIRT6 by ubiquitin-specific peptidase (USP10) can stabilize SIRT6 and protect against diet-induced hepatic steatosis [74]. 

SIRT6 also plays a significant role in the control of total and LDL cholesterol. SIRT6 coordinates with FOXO3 for the regulation of cholesterol biosynthesis by the suppression of the SREBP2 gene transcription through histone H3K9 and H3K56 deacetylation. Sirt6 LKO mice exhibit elevated cholesterol levels in the blood and liver. Hepatic SIRT6 overexpression improves hypercholesterolemia in db/db mice [75]. Another study using HepG2 cells also suggests the repression of SREBP1 and SREBP2 by SIRT6 at three different levels: transcriptional repression, protein maturation inhibition, and SREBP1 phosphorylation by AMP-activated protein kinase (AMPK) [76]. In addition to the control of the total cholesterol levels, SIRT6 also modulates LDL-cholesterol levels through the suppression of the transcription of the proprotein convertase subtilisin/kexin type 9 (PCSK9) gene. At the molecular level, SIRT6 is recruited by FOXO3 to the PCSK9 gene promoter region and deacetylates H3K9 and H3K56, and at the same time, HNF1 homeobox A (HNF1A), a transcriptional activator, is excluded from the PCSK9 gene promoter [77]. Sirt6 LKO mice have significantly elevated LDL/VLDL—but not high-density lipoprotein (HDL)-cholesterol levels compared to WT mice. VLDL secretion is also increased in Sirt6 LKO mice as compared to that in WT mice. Adenoviral SIRT6 overexpression normalizes serum LDL-cholesterol levels in a high-fat diet-induced hypercholesterolemia mouse model [77].

## 3. SIRT6 in Hepatic Fatty Acid Oxidation (FAO)

**Figure 4:**
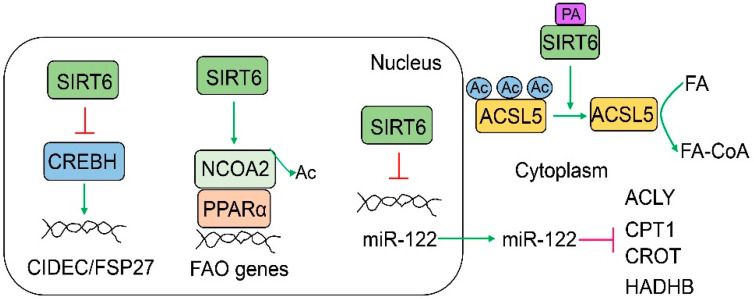
**SIRT6 promotes fatty acid oxidation by multiple mechanisms.** In the nucleus, SIRT6 can induce FAO genes through the regulation of NCOA2 and PPARα or indirectly promotes FAO through the inhibition of the CREBH transcriptional activation of the CIDEC gene or the miR-122 gene transcription. In the cytoplasm, palmitate-bound SIRT6 can deacetylate ACSL5 to directly increase the ACSL5 enzymatic activity by converting long-chain fatty acids to long-chain fatty acyl-CoAs.

In addition to the suppression of hepatic lipogenesis, SIRT6 increases hepatic fatty acid oxidation. SIRT6 promotes fatty acid oxidation in the liver through several mechanisms (Figure 4). In the nucleus, SIRT6 can suppress the expression of cell death inducing DFFA-like effector C (CIDEC) gene, encoding a lipid droplet-associated protein that promotes lipid droplet enlargement and inhibits lipolysis. By the repression of the CIDEC transcription, SIRT6 promotes fasting- or ketogenic diet-induced ketogenesis [78]. SIRT6 also directly induces FAO gene expression through the regulation of nuclear receptor coactivator 2 (NCOA2) and peroxisome proliferator activated receptor alpha (PPARα). Mechanistically, SIRT6 deacetylates NCOA2 at K780 and enhances the coactivation activity of NCOA2 [48]. SIRT6 also promotes FAO gene expression indirectly by the downregulation of microRNA-122 (miR-122). It has been shown that the expression of several FAO genes including hydroxyacyl-CoA dehydrogenase trifunctional multienzyme complex subunit β (HADHB), carnitine palmitoyltransferase 1 (CPT1), carnitine O-octanoyltransferase (CROT), and ATP citrate lyase (ACLY) is decreased by the overexpression of miR-122 but increased by the overexpression of miR-122 antagomir; however, it is unclear whether those gene transcripts are direct targets of miR-122 or not. There is another layer of complication: SIRT6 and miR-122 reciprocally regulate each other at transcriptional and posttranscriptional levels, respectively [79]. Interestingly, when cellular saturated fatty acids (short-chain, medium-chain, and long-chain), especially palmitic acids, are elevated, SIRT6 is transported to cytoplasm by exportin 2 and activates an FAO enzyme acyl-CoA synthetase long-chain family member 5 (ACSL5). SIRT6 deacetylates ACSL5 at three major lysine residues, K98, K361, and K367, and the deacetylated ACSL5 is more active for the promotion of FAO [16]. Interestingly, unsaturated fatty acids do not have such an effect on SIRT6. The overexpression of deacetylated ACSL5 mutant (K98R/K361R/K367R) in the liver of Sirt6 LKO mice significantly improves high-fat diet-induced glucose intolerance and fatty liver. SIRT6 protein levels are decreased and ACSL5 K361 acetylation levels are increased in the liver of nonalcoholic steatohepatitis (NASH) patients compared to those in healthy controls [16].

## 4. SIRT6 in Acute Liver Injury

**Figure 5:**
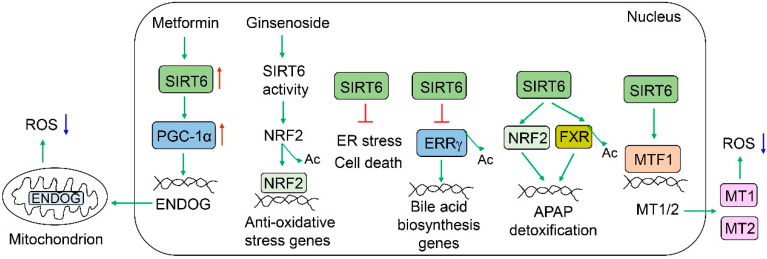
**SIRT6 protects against liver injury.** SIRT6 has multiple mechanisms for defending against oxidative stress, including the induction of ENDOG and MT1/2 and the activation of NRF2. SIRT6 can activate FXR and NRF2 to alleviate APAP-induced liver injury. Intrahepatic bile acid accumulation may cause cholestasis. SIRT6 can suppress ERRγ-mediated bile acid biosynthesis to reduce bile acid-induced liver injury.

SIRT6 plays an important role in the protection against various liver injuries (Figure 5). Alcohol metabolism in the liver produces reactive oxygen species (ROS). SIRT6 has been shown to protect against alcohol-induced liver injury, as Sirt6 LKO mouse livers exhibit increased oxidative stress and liver injury, and Sirt6 liver-specific transgenic mice are protected from alcohol-induced liver injury. Hepatic SIRT6 protein levels are significantly decreased in the livers of alcoholic cirrhotic patients and alcoholic diet-fed mice (5% ethanol for 4 weeks or 6% ethanol for 15 days plus a single binge) [80]. Sirt6 LKO mice are much more susceptible to an alcoholic diet compared to WT mice, as manifested by higher serum alanine aminotransferase (ALT) levels, elevated hepatic triglycerides and cholesterol, and increased ROS and lipid peroxidation [80]. One of the defense mechanisms employed by SIRT6 is the induction of metallothioneins (MT1/2) through the coactivation of metal regulatory transcription factor 1 (MTF1) [80]. The adenoviral overexpression of MT1 significantly ameliorates ethanol diet-induced liver injury and alcohol-related liver disease (ALD) in Sirt6 LKO mice. Hepatic Sirt6 overexpression also protects mice from ethanol-induced liver injury and ALD [80]. In addition, SIRT6 can also activate NFE2-like BZIP transcription factor 2 (NFE2L2, commonly known as NRF2) by deacetylation to ameliorate alcohol-associated liver injury, and ginsenoside Rc, a major active ingredient of ginseng, can protect from ALD by the activation of the SIRT6-NRF2 defense axis [53]. SIRT6 also improves alcohol-induced liver damage by dampening endoplasmic reticulum (ER) stress [81]. Hepatic Sirt6 deficiency exacerbates ethanol-induced ER stress in the liver with elevated protein levels of DNA damage inducible transcript 3 (DDIT3, also named CHOP), heat shock protein family A member 5 (HSPA5, also named BiP), activating transcription factor 4 (ATF4), and XBP1. Hepatic Sirt6 overexpression significantly ameliorates the ethanol-induced ER stress in the liver [81]. SIRT6 also reduces high-fat diet-induced oxidative stress by the induction of PPARG coactivator 1 alpha (PGC-1α) and endonuclease G (ENDOG) [82]. Upon exposure to a high concentration of acetaminophen (APAP), SIRT6 is induced by tumor protein p53 (TP53) activation and subsequently activates NRF2 and farnesoid X receptor (FXR) for protection against APAP-induced liver injury [38,83]. Regarding the regulation of FXR, SIRT6 deacetylates and activates FXR transcriptional activity. Some of the FXR target genes such as ATP binding cassette subfamily B member 11 (ABCB11, also named BSEP) and glutamate-cysteine ligase catalytic subunit (GCLC) contribute to APAP excretion/detoxification and anti-oxidative stress, respectively [38]. Bile acid accumulation (cholestasis) can cause liver damage. It has been shown that Sirt6 LKO mice are more susceptible to bile duct ligation (BDL)-induced liver injury than their wild-type counterparts, and the adenoviral overexpression of Sirt6 in the liver remarkably protects mice from BDL-induced liver injury and fibrosis. At the molecular level, SIRT6 deacetylates and destabilizes estrogen-related receptor gamma (ERRγ), a transcriptional activator for bile acid production in the liver. As a result, ERRγ is degraded through the ubiquitin-proteasome system, and hepatic bile acid biosynthesis is attenuated [26]. SIRT6 is also implicated in ischemia/reperfusion-induced liver injury. Sirt6 LKO mice exhibit more oxidative stress, mitochondrial damage, and inflammation than WT counterparts [84].

## 5. SIRT6 in Hepatic Inflammation

**Figure 6:**
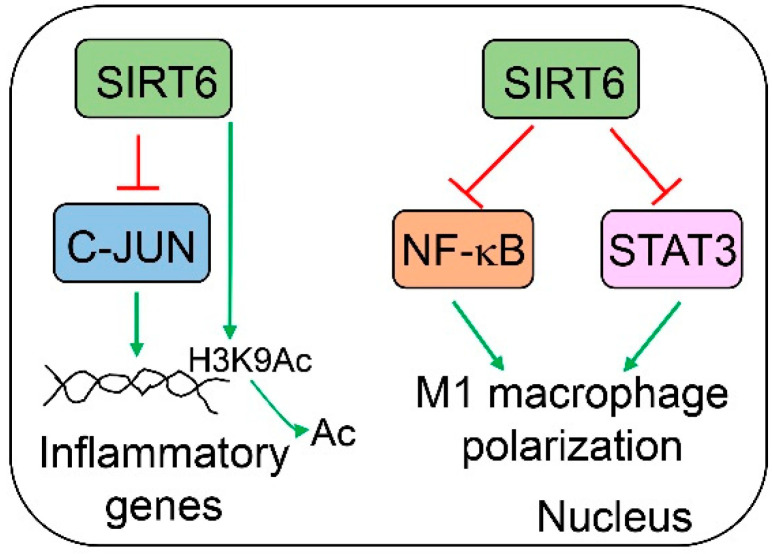
**SIRT6 modulates hepatic inflammation.** Specifically, SIRT6 can suppress M1 macrophage polarization by the inhibition of NF-κB and STAT3 activities and represses c-JUN-mediated inflammatory genes.

Sirt6 plays a significant role in the modulation of immune cells, especially macrophages (Figure 6). Sirt6 null mice that survive from hypoglycemia suffer progressive hepatic inflammation starting at 2 months of age. Leukocytes are infiltrated in the entire liver of the Sirt6 whole-body knockout mice. The majority of the infiltrated inflammatory cells are CD3-positive. A significant number of inflammatory cells are also positive for F4/80 (macrophage marker) and myeloperoxidase (MPO, a neutrophil marker). By comparing phenotypes in T cell-specific (Lck-Cre) and myeloid-derived cell-specific (Lyz2-Cre) Sirt6 knockout mice, hepatic inflammation is more pronounced in the later model than the former one, suggesting a stronger role of myeloid Sirt6 in the modulation of hepatic inflammation [85]. Bone marrow-derived Sirt6-deficient macrophages exhibit an increased production of interleukin 6 (IL6), C-C motif chemokine ligand 2 (CCL2, also called MCP1), and TNFα under non-stimulated conditions. At the molecular level, SIRT6 interacts with and suppresses Jun proto-oncogene (c-JUN) at the promoter regions of the IL6 and CCL2 genes. At the same time, SIRT6 also deacetylates H3K9 in the promoter chromatin of those target genes [85]. The role of Sirt6 in macrophages is further confirmed by another laboratory under a high-fat diet condition. Myeloid-specific Sirt6 knockout mice exhibit worse systemic insulin resistance and glucose intolerance, heavier liver weights, higher hepatic triglyceride and cholesterol levels, higher hepatic macrophage infiltration, and higher nonalcoholic fatty liver disease (NAFLD) activity scores than their WT counterparts after 16 weeks of high-fat diet feeding [86]. The expression of M1 macrophage genes including F4/80, CD11c, CD11b, CCL2, C-C motif chemokine receptor 2 (CCR2), TNFα, IL6, IL1b, and intercellular adhesion molecule 1 (ICAM1) is increased, whereas M2 macrophage genes such as arginase 1 (ARG1) and IL10 are downregulated. Epididymal white adipose tissue in Sirt6 myeloid-specific knockout mice also exhibits increased M1 macrophage infiltration. At the molecular level, Sirt6 deficiency induces multiple inflammatory pathways, including c-JUN, nuclear factor of kappa light polypeptide gene enhancer in B cells (NF-κB), and signal transducer and activator of transcription 3 (STAT3). Those molecular changes also push macrophages to M1 polarization [85,86]. Bone marrow-derived macrophages from Sirt6 myeloid-specific knockout mice display higher responses to lipopolysaccharide (LPS), with an elevated activation of p38 mitogen-activated protein kinase (MAPK, but not c-JUN N-terminal kinase, JNK, or extracellular signal-regulated kinase, ERK), NF-κB, and STAT3 compared to the WT counterpart. Blocking NF-κB, the inhibition of STAT3, or the antagonism of the IL-6 receptor can significantly suppress M1 macrophage polarization. The hyperacetylation of pyruvate kinase M2 (PKM2) in Sirt6-deficient macrophages also plays a significant role in the regulation of STAT3 activation and M1 macrophage polarization [86].

## 6. SIRT6 in Hepatic Fibrosis

**Figure 7:**
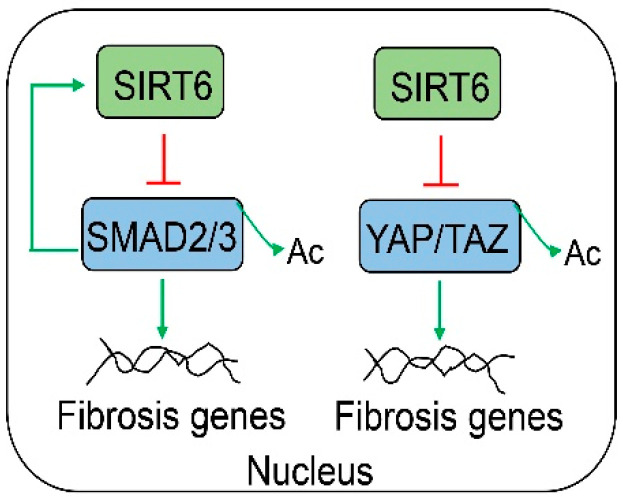
**SIRT6 has an anti-fibrosis function.** SIRT6 can control hepatic fibrosis by the suppression of the TGFβ-SMAD and YAP/TAZ pathways through deacetylation.

Liver injury and inflammation can trigger hepatic fibrosis chronically. Hepatic stellate cells (HSCs) are believed to be a major contributor to liver fibrosis. In the mouse liver, Sirt6 is abundantly expressed in HSCs [11,13]. During the process of HSC activation, Sirt6 is markedly decreased. In diet- or chemical-induced liver fibrosis mouse models, hepatic Sirt6 protein levels are also significantly decreased compared to those in normal animals [11,13]. HSC-specific Sirt6 knockout mice are more susceptible to diet- (high-fat and high-cholesterol) or chemical (CCl_4_ or DDC: 5-diethoxycarbonyl-1,4-dihydrocollidine)-induced liver fibrosis than WT mice [11,13]. Two major signaling pathways have been implicated in the regulation of HSC activation by SIRT6 (Figure 7). The transforming growth factor beta (TGFβ)-SMAD signaling has been thought to be one of the most potent pathways for liver fibrosis. Not surprisingly, SIRT6 strongly attenuates the SMAD action by the suppression of both SMAD2 and SMAD3 [11,13,87]. SIRT6 deacetylates SMAD2 (K54) and SMAD3 (K333 and K378) to dampen their transcriptional activities [11,13]. Interestingly, TGFβ signaling also induces SIRT6 gene transcription through the activation of SMAD3 [88]. In addition, YAP and TAZ/WWTR1 have also been implicated in liver fibrosis [89]. TAZ can be activated by Indian hedgehog ligands to promote HSC activation. The knockdown of TAZ by small interference RNAs has been shown to improve hepatic fibrosis in a diet-induced NASH mouse model. YAP is also activated in fibrotic livers of human patients infected with hepatitis C virus or CCl_4_-treated mice. SIRT6 can suppress the coactivator activities of YAP and TAZ by deacetylation [12]. In human LX-2 hepatic stellate cells, SIRT6 overexpression decreases YAP and TAZ acetylation and increases YAP Ser127 phosphorylation. In contrast, YAP and TAZ acetylation levels are significantly increased in the mouse primary HSCs from Sirt6 HSC-specific knockout mice compared to those of WT mice. It has been shown that SIRT6 directly interacts with YAP and TAZ and deacetylates them at multiple lysine residues. Specifically, the deacetylation of K102 in human YAP1 has a remarkable suppressive effect on YAP1 transcriptional coactivation activity. Similarly, the deacetylation of K39 in mouse Taz has a significant repressive effect on Taz coactivation activity. In addition, by an interaction of YAP or TAZ, SIRT6 also alters their protein complex formation with a decrease in the YAP/TAZ-TEAD1 (TEA domain transcription factor 1) activation complexes but an increase in the TEAD1-VGLL4 (vestigial-like family member 4) repression complexes [12]. A recent report also suggests crosstalk from hepatocyte SIRT6 to HSCs. Human centenarian-associated SIRT6 variants (N308K/A313S) have significant effects on hepatocyte metabolome and secretome. The overexpression of these SIRT6 variants in immortalized human hepatocytes suppresses collagen deposition and fibrosis gene expression in a spheroid model with the coculture of hepatocytes and HSCs [90]. In a bile duct ligation-induced cholestatic mouse model, Sirt6 LKO mice manifest more severe liver injury and fibrosis than WT controls, partly due to elevated ERRγ activity, as ERRγ is an SIRT6 deacetylase substrate [26].

## 7. SIRT6 in Liver Cancer

**Figure 8:**
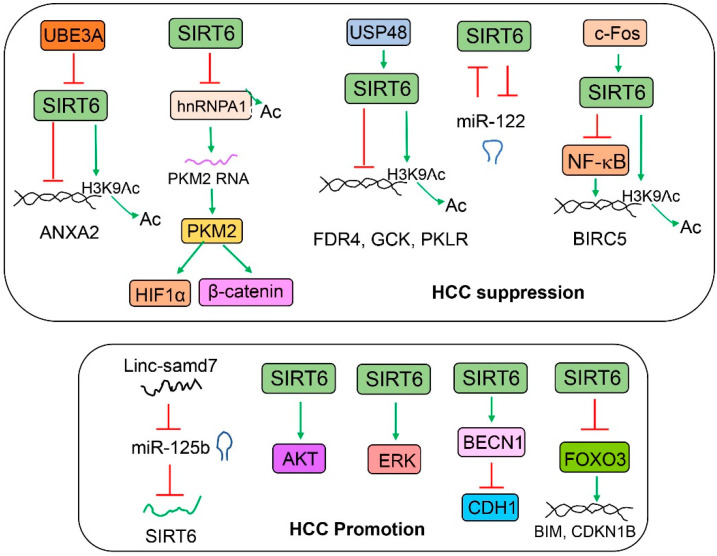
**The role of SIRT6 in liver cancer.** The top panel describes the anti-cancer functions of SIRT6. SIRT6 can suppress tumor-promoting factors through either gene repression by the deacetylation of histone H3 or the suppression of an intermediate factor such as hnRNPA1. The bottom panel describes the tumor-promoting activities of SIRT6. AKT and ERK have been shown to be activated by SIRT6, although the mechanisms remain unclear. In some conditions, SIRT6 may also inhibit some tumor suppressors such as CDH1 and FOXO3.

SIRT6 has been implicated in liver cancer either favorably or unfavorably (Figure 8). In one study, when Sirt6 LKO and WT mice were administered with a single dose of DEN (25 mg/kg) at 14 days of age and CCl_4_ (10%, 8 mL/kg, intraperitoneally, IP) for 14 weeks (twice/week) as of 8 weeks, Sirt6 LKO male mice developed three times more liver tumor nodules than their WT counterparts at the end of the experiment. Sirt6 overexpression reduced tumor growth in a xenograft mouse model [91]. In another study, Sirt6 has been shown to suppress hepatoblastoma (HepG2 and Huh6) cell viability and invasion through the transcriptional repression of Wnt receptor frizzled 4 (FDR4). Interestingly, quercetin is found to induce SIRT6 gene expression and suppress HepG2 and Huh6 cell viability and migration [28]. SIRT6 protein can be stabilized by ubiquitin-specific peptidase (USP48)-mediated deubiquitination at the K33 and K128 residues of SIRT6. As a result, SIRT6 suppresses hepatocellular carcinoma (HCC) growth through the inhibition of glycolysis [92]. It has been shown that SIRT6 reduces aerobic glycolysis and the cell proliferation of HepG2 and Huh7 cells by the deacetylation and inhibition of heterogeneous nuclear ribonucleoprotein A1 (hnRNPA1) for the alternative splicing of PKM2 [43]. SIRT6 can suppress ubiquitin protein ligase E3A (UBE3A)-mediated liver tumor growth by the epigenetic repression of annexin A2 (ANXA2) in HepG2 cells [93]. miR-122 has been shown to be a tumor suppressor of hepatocellular carcinoma. Interestingly, SIRT6 and miR-122 reciprocally regulate each other. In the Cancer Genome Atlas (TCGA) datasets, hepatocellular carcinoma patients who did not have a negative correlation between SIRT6 and miR-122 expression had a better prognosis than those who had a negative correlation [79]. SIRT6 has been shown to suppress HepG2 cell viability and growth partly through the inhibition of ERK phosphorylation and the reduction in oxidative stress [94]. In an analysis of the Oncomine Cancer Microarray database containing 153 primary human liver cancers and cirrhotic and normal livers, SIRT6 mRNA levels were found to be significantly lower in the liver cancers and cirrhotic livers than they were in the normal livers. Gene expression analysis of Sirt6 knockout hepatocytes also reveals the elevated expression of HCC biomarkers including alpha-fetoprotein (AFP), insulin-like growth factor 2 (IGF2), H19, and glypican 3 [95]. Another study suggests that SIRT6 plays a key tumor suppression function during the liver cancer initiation. SIRT6 protein is decreased in human dysplastic liver nodules but not in malignant liver tumors. The suppression of survivin (also named baculoviral IAP repeat-containing 5, BIRC5) by SIRT6 is proposed as a potential mechanism [96].

A number of reports also suggest that SIRT6 may also be involved in the promotion of liver cancer. SIRT6 has been reported to mediate the effect of long intergenic noncoding RNA smad7 (Linc-smad7) on hepatocellular carcinoma proliferation and migration in Hep3B and Huh7 cell lines [97]. In another report, SIRT6 has been shown to mediate the tumor-promoting effect of NAD(P)H:quinone oxidoreductase 1 (NQO1) on hepatocellular carcinoma through the activation of AKT serine/threonine kinases in PLC/PRF/5 and Huh7 cell lines and an orthotopic tumor cell implantation mouse model [98]. SIRT6 has been reported to decrease E-cadherin (encoded by the CDH1 gene) in normal or hepatocellular carcinoma cell lines through the deacetylation of Beclin 1, a key regulator of autophagy, and the promotion of autophagy-mediated E-cadherin degradation [17]. It has been shown that SIRT6 overexpression increases proliferation and decreases apoptosis in Huh7 cells, partly through the activation of ERK phosphorylation, an increase in the expression of BCL2 apoptosis regulator (BCL2), and a decrease in expression of BCL2-associated X apoptosis regulator (BAX) [99,100]. SIRT6 overexpression also reduces doxorubicin-induced cell death in HepG2 and Huh7 cells, partly through the regulation of FOXO3 stability and nuclear translocation [101]. SIRT6 can also reduce BAX-mediated apoptosis in Huh7 and SK-Hep-1 cells, partly through the deacetylation of Ku70 protein (encoded by the XRCC6 gene, X-ray repair cross complementing 6) at the K542 residue [44]. SIRT6 has also been shown to promote hepatocellular carcinoma growth through the inhibition of cell senescence and cell growth arrest [102,103].

## 8. SIRT6 Chemical Modulators

As SIRT6 has been implicated in multiple diseases including fatty liver disease and liver cancer, a number of chemical modulators (activators or inhibitors) targeting SIRT6 have been developed. Multiple pyrrolo [1,2-α]quinoxaline-based derivatives have been developed, and several of them (#35, 36, 38, 46, 47, and 50) exhibited the selective activation of SIRT6 but not SIRT1, SIRT2, SIRT3, or SIRT5. Some of these SIRT6 activators showed an anti-inflammatory effect on LPS-treated BV2 microglial cells. Interestingly, some of the compounds also exhibited anti-SARS-CoV-2 activities in a luciferase reporter assay [104]. Atractylenolide I (ATL I) was predicted to bind to SIRT6 by molecular docking, and it was later confirmed to activate SIRT6, as evidenced by a decrease in H3K9 and H3K56 acetylation levels in mouse primary hepatocytes and livers. Sirt6 knockout abolished the anti-fatty liver effect of ATL I in high-fat diet-treated mice [105]. Nitro-fatty acids including nitro-oleic acid and nitro-conjugated linoleic acid have been shown to activate the deacetylase activity of SIRT6 through binding to the hydrophobic crevice of the SIRT6 active site [106]. Galloflavin and ellagic acids, the most common polyphenols in berries, have been shown to activate the deacetylase activity of SIRT6, possibly through a direct interaction with G6 and D188 residues [107]. Structure–activity relationship analysis has led to the identification of the 2-(1-benzofuran-2-yl)-*N*-(diphenylmethyl) quinoline-4-carboxamide (also named 12q) compound as a very potent SIRT6 activator with an EC_50_ of 5.35 ± 0.69 µM in an in vitro deacetylase assay. Interestingly, this compound also showed a significant inhibition of pancreatic ductal adenocarcinoma tumor growth [108]. Scaffold-based screens (molecular docking) and chemical optimization have led to the development of MDL-800 and MDL-801 compounds as potent SIRT6 allosteric activators with EC_50_ values of 10.3 ± 0.3 µM and 5.7 ± 0.3 µM in the in vitro deacetylase assays, respectively. Interestingly, MDL-800 exhibits a marked inhibition of HCC tumor cell growth in cell line and xenograft mouse models [109]. Further structure-based modifications have allowed for the identification of MDL-811 as a much more potent activator, with an EC_50_ of 5.7 ± 0.8 µM and enhanced bioavailability (F%, 92.96%). Remarkably, MDL-811 also exhibited a significant tumor suppressive effect against colorectal cancer in cell lines and xenograft mouse models [110]. Long-chain fatty acids including myristic, palmitic, stearic, oleic, and linoleic acids have been shown to activate SIRT6 deacetylase activity in vitro [111]. Oleoylethanolamide has also been shown to activate SIRT6 catalytic activity [112]. Through a screen of fatty acid and bioactive lipid libraries and chemical modification, 2-(3-chloro-4-(2,4,6-trichloro-*N*-(2,4,6-trichlorobenzoyl)benzamido)phenyl)-1,3-dioxoisoindoline-5-carboxylic acid (CL5D) has been shown to be a potent SIRT6 activator [113]. By using a very robust mass-spectrometry-based assay, quercetin and luteolin are shown to have low potency of the activation of SIRT6 deacetylation. Cyanidin, another quercetin derivative, exhibits a higher potency than quercetin on the SIRT6 deacetylase activation. Isoquercetin has a lower potency but a higher specificity for the activation of SIRT6 deacetylation. However, catechin gallate, the most potent quercetin derivative, has an inhibitory effect on SIRT6 deacetylation [114]. By the chemical modifications of a quinoxaline scaffold, 4-(pyridine-3-yl)-4,5-dihydropyrrolo[1,2-α]quinoxaline (also called UBCS039) has been found to be a relatively specific SIRT6 activator, with an EC_50_ of 38 ± 13 µM in an in vitro assay [115].

A number of SIRT6 inhibitors have also been reported to date. By screening 1-phenylpiperazine derivatives, 5-(4-methylpiperazin-1-yl)-2-nitroaniline (also called 6d) has been found to be a potent inhibitor against SIRT6 deacetylase activity, with an IC_50_ of 4.93 µM in an in vitro deacetylation assay. This compound also showed very good selectivity against SIRT6 but not SIRT1-3 or HDAC1-11 (up to 200 µM of 6d) [116]. Trichostatin A, a well-known inhibitor of class I and class II HDACs, has also been shown to inhibit the deacetylase activity of SIRT6 but not other sirtuins at low micromolar concentrations, in contrast to a partial inhibition by low millimolar concentrations of nicotinamide [117]. Several salicylate-derived compounds have been shown to inhibit SIRT6 deacetylase activity in vitro and in cells. These compounds exhibit the growth inhibition of T lymphocytes and the sensitization of the pancreatic cancer cell-killing effect of gemcitabine (a nucleoside analog) [118]. Based on a quinazolinedione-like scaffold, several analogs have been found to have an inhibitory effect on SIRT6 deacetylase activity. Compounds 2, 3, and 8 have been shown to sensitize pancreatic cancer cells to gemcitabine and olaparib (a PARP inhibitor) [119]. Thiomyristoyl peptides have been shown to be cell-permeable inhibitors against SIRT6, although not very specifically [120]. Structure-based compound screens have identified several inhibitors of SIRT6, and one of them (compound 9) has relative selectivity for SIRT6 versus SIRT1 and SIRT2 [121].

## 9. Concluding Remarks

As an epigenetic regulator, SIRT6 has been demonstrated to be a salutary factor in hepatic metabolic homeostasis and liver health. SIRT6 reduces triglycerides, cholesterol, and LDL in hepatocytes, controls macrophage polarization, and suppresses HSC activation and fibrogenesis. These functional characteristics indicate SIRT6 to be a favorable target for the prevention or treatment of ALD, NAFLD, and NASH. As SIRT6 has a strong anti-oxidative stress function, boosting SIRT6 activity may ameliorate alcohol- or drug-induced liver injury. As the role of SIRT6 in liver cancer remains incompletely understood, further characterization of the SIRT6 function in liver cancer development is needed. Additionally, translational and clinical studies are needed to shed light on the therapeutic potentials of SIRT6.

## Figures and Tables

**Table 1:** Known substrates of SIRT6 deacetylase, deacylase, and mono-ADP ribosyltransferase activities.

Substrate Protein Names	Specific Residue(s)	References
**Deacetylase Substrates**		
ACSL5, acyl-CoA synthetase long-chain family member 5	K98, K361, K367	[16]
BECN1, beclin 1	Not determined	[17]
CAV1, caveolin 1	Not determined	[18]
CDH1, cadherin 1	K135	[19]
CHREBP/MLXIPL, carbohydrate response element binding protein	K672	[20]
CTNNB1, catenin beta 1	Not determined	[21]
DDB2, damage-specific DNA binding protein 2	K35, K77	[22]
DNMT1, DNA methyltransferase 1	Not determined	[23]
ELF5, E74-like ETS transcription factor 5	Not determined	[24]
ERα/ESR1, estrogen receptor 1	K171, K299	[25]
ERRγ/ESRRG, estrogen-related receptor gamma	K195	[26]
EZH2, enhancer of Zeste 2 polycomb repressive complex 2 subunit	Not determined	[27]
FZD4, frizzled class receptor 4	Not determined	[28]
FOXO1, forkhead box O1	Not determined	[29,30,31,32,33,34,35,36]
FOXO3, forkhead box O3	K242, K245	[31,37]
FXR/NR1H4, farnesoid X receptor	Not determined	[38]
GATA3, GATA binding protein 3	Not determined	[39]
GCN5/KAT2A, lysine acetyltransferase 2A	K549	[14]
HIF1A, hypoxia inducible factor 1A	Not determined	[40]
Histone H3	K9, K18, K56	[6,7,8,41]
HMGB1, high-mobility group box 1	Not determined	[42]
HNRNPA1, heterogenous nuclear ribonucleoprotein A1	K3, K52, K87, K350	[43]
Ku70/XRCC6, X-ray repair cross complementing 6	Not determined	[44,45]
LXR/NR1H3, liver X receptor	K432	[20]
MRTFA, myocardin-related transcription factor A	Not determined	[46]
NAMPT, nicotinamide phosphoribosyltransferase	Not determined	[47]
NCOA2, nuclear receptor coactivator 2	K780	[48]
NFATC4, nuclear factor of activated T cells 4	Not determined	[49]
NF-κB/RELA, NF-kappa-B transcription factor	K310	[50,51]
NOS3, nitric oxide synthase 3	K494, K497, K504	[52]
NRF2/NFE2L2, NFE2-like BZIP transcription factor 2	Not determined	[53]
P53/TP53, tumor protein P53	K381	[54]
PER2, period circadian regulator 2	Not determined	[55]
PKM2, pyruvate kinase M2	K433	[56]
RBBP8, RB binding protein 8	Not determined	[57]
RUNX2, RUNX family transcription factor 2	Not determined	[58]
SMAD2, SMAD family member 2	K54	[13]
SMAD3, SMAD family member 3	K333, K378	[11]
SREBP1/SREBF1, sterol regulatory element binding protein 1	K289	[20]
STAT3, signal transducer and activator of transcription 3	K685	[59]
STAT5, signal transducer and activator of transcription 5	K163	[60]
TAZ/WWTR1, WW domain-containing transcription regulator 1	K39, K54	[12]
TAU/MAPT, microtubule-associated protein Tau	K174	[61]
TRF2, telomeric repeat binding factor 2	K176, K179, K190	[62]
XBP1, X-box binding protein 1	K257, K297	[15]
YAP1, Yes1-associated transcriptional regulator	K76, K90, K97, K102, K440	[12]
**Deacylase substrates**		
RRAS2, RAS-related 2	K192, K194, K196, K197	[10]
TNFα, tumor necrosis factor alpha	K19, K20	[9]
**Mono-ADP ribosyltransferase substrates**		
BAF170/SMARCC2, BRG1-associated factor 170	K312	[63]
KAP1/TRIM28, tripartite motif-containing 28	Not determined	[64]
KDM2A, lysine demethylase 2A	R1020	[65]
LMNA, lamin A/C	Not determined	[66]
PARP1, poly(ADP-ribose) polymerase 1	K521	[67]
SIRT6, sirtuin 6	Not determined	[5]

## Data Availability

No new data were generated.
